# Cytotoxicity and anti-HIV activities of extracts of the twigs of *Croton dichogamus* Pax

**DOI:** 10.1186/s12906-022-03532-1

**Published:** 2022-02-25

**Authors:** Ermias Mergia Terefe, Faith Apolot Okalebo, Solomon Derese, Gaber El-Saber Batiha, Amal Youssef, Mohammed Alorabi, Joseph Muriuki

**Affiliations:** 1 Department of Pharmacology and Pharmacognosy, School of Pharmacy and Health Sciences, United States International University-Africa, Nairobi, Kenya; 2 Department of Pharmacology and Pharmacognosy, College of Health Sciences, University of Nairobi, Nairobi, Kenya; 3 Department of Chemistry, University of Nairobi, Nairobi, Kenya; 4 Department of Pharmacology and Therapeutics, Faculty of Veterinary Medicine, Damanhour University, AlBeheira, Egypt; 5 Department of Medical Pharmacology, Faculty of Medicine, Cairo University, Giza, Egypt; 6 Department of Biotechnology, College of Sciences, Taif University, Taif, Saudi Arabia; 7 Centre for Virus Research, Kenya Medical Research Institute, Nairobi, Kenya

**Keywords:** HIV, Croton dichroism, Cytotoxicity, Anti-viral activity

## Abstract

**Background:**

Acquired immunodeficiency syndrome (AIDS) is a clinical syndrome resulting from infection with human immunodeficiency virus (HIV), which causes profound immunosuppression. Anti-HIV drugs that are currently available are chemically synthesized and are frequently limited by side effects, the emergence of drug resistance, affordability, and availability, with over 5 million people in the world lacking access to treatment. As a result, to discover new anti-HIV agents, we investigated the effects of Kenyan C. dichogamus extracts on the laboratory-adapted strain HIV-1_IIIB_ in human T-lymphocytic MT-4 cells.

**Methods:**

Four soluble fractions of 1:1 *v/v* CH_2_Cl_2_:MeOH extract of the twigs of *C. dichogamus* Pax were tested for their replication inhibition activity against the laboratory-adapted strain HIV-1_IIIB_ in the human T-lymphocytic MT-4 cell line. The plant extracts were further evaluated for their cytotoxicity in MT-4 cells using the MTT assay.

**Results:**

The cytotoxicity CC_50_ values of the methanol and methylene chloride soluble fractions of *C. dichogamus* were found to be between 19.58 ± 0.79 and 167 ± 0.8 µg/ml, respectively. The hexane, methylene chloride, and methanol soluble fractions of the 1:1 *v/v* CH_2_Cl_2_:MeOH extract of the twigs of *C. dichogamus* showed inhibition of the HIV-1_IIIB_ laboratory-adapted strain in a virus-infected cell culture antiviral assay. The methanol soluble fraction of the 1:1 *v/v* CH_2_Cl_2_:MeOH extract of the twigs of *C. dichogamus* showed significant anti-HIV activity by inhibiting more than 90% of viral-induced cytopathic effects with an IC_50_ value of 0.06 ± 0.01 µg/ml, giving an SI of 318.5.

**Conclusion:**

Based on our findings, the methanol soluble fraction of the 1:1 *v/v* CH_2_Cl_2_:MeOH extract of the twigs of *C. dichogamus* has shown potential efficacy in inhibiting viral replication and could be considered a promising candidate for further studies.

## Background

Acquired immunodeficiency syndrome (AIDS) is one of the most severe diseases, with approximately 38 million people infected with human immunodeficiency virus (HIV-1) [1]. Infection with HIV-1 has been identified as the causative agent of AIDS. There have been significant advances in rational drug design and synthesizing highly active compounds [2]. Although HIV drug resistance and side effects are becoming increasingly common, as well as the need for long-term antiviral treatment, the development of new anti-HIV agents is becoming increasingly necessary [3]. To prevent disease progression, anti-HIV drugs with minimal toxic effects, which target different metabolic pathways of HIV infection, should prove beneficial. It appears that searching for natural substances may be a more effective strategy for discovering novel antivirals with lower cytotoxicity (s) [4].

Natural products are still regarded as the richest source of bioactive compounds because of their abundance. When discovering anti-HIV treatments, natural products promote an abundance of availability while also containing a massive amount of potentially bioactive compounds for successful drug discovery. Many natural products have been reported to have significant anti-HIV activity. Many of these compounds have been shown to be effective at inhibiting HIV-1 activity at almost every stage of the viral life cycle [5]. Several plant-derived compounds, including alkaloids, coumarins, carbohydrates, flavonoids, lignans, phenolics, quinines, phospholipids, terpenes, and tannins, have been identified and reported to have inhibitory activity against various targets during the viral life cycle of HIV.

**Fig. 1:**
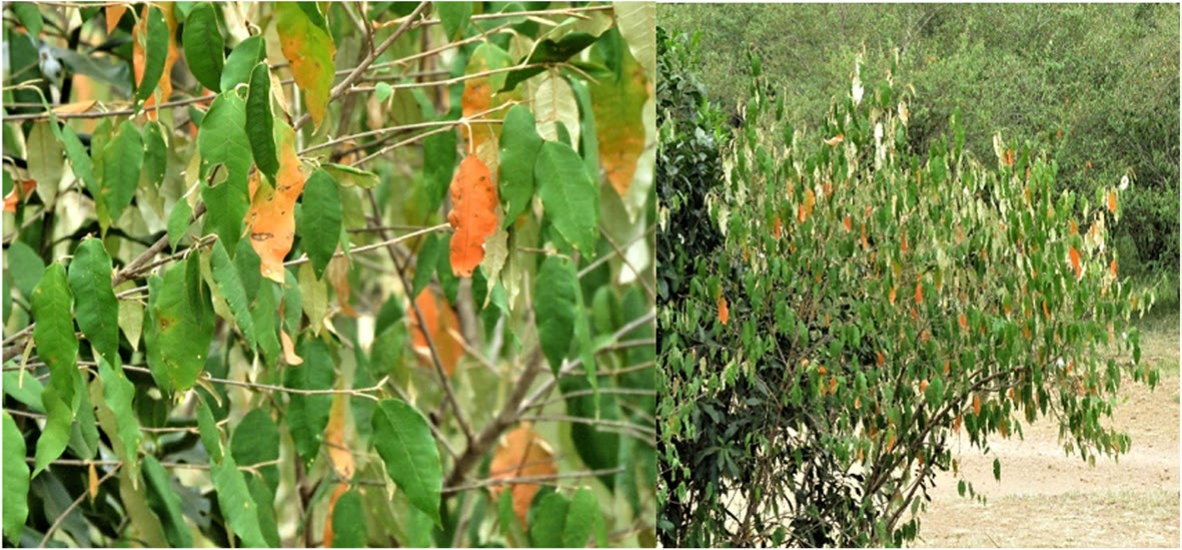
Photo of *Croton dichogamus* Pax *(Euphorbiaceae)* plant. (Photo by Ermias M Terefe)

African traditional medicine is untapped, yet many plants have been used for eons as antiviral agents. Several plants of the expansive *Croton* genus have been reported to have anti-HIV activities, including *C. echinocarpus* [6], *C. megalobotrys* [7]*,* and *C. tiglium* [8]*.* Therefore, to find new anti-HIV agents, we studied extracts of Kenyan *C. dichogamus* for their inhibitory effects against the laboratory-adapted strain HIV-1IIIB in human T-lymphocytic MT-4 cells. *In East African countries, C. dichogamus* (Fig. 1*)* has wide ethnomedicinal use, including fever, stomach illness, respiratory disorders, malaria, impotence, and infertility [9–13]. The objective of this study was to evaluate the cytotoxicity and anti-HIV activity of the methanol soluble fraction of the 1:1 *v/v* CH_2_Cl_2_/MeOH extract of twigs of *C*. *dichogamus* using an in vitro model.

## Materials and methods

### Preparation of crude extract

The twigs of *C. dichogamus* were collected in June 2020 from the Mwala Constituency in Muthetheni, Machakos County, Kenya (S 1º 28ʹ 60ʹʹ, E 37º 30ʹ 02ʹʹ). Ethical approval to collect *C. dichogamus* was obtained from the Kenyatta National Hospital-University of Nairobi Ethics and Research Committee (KNH-UON ERC) with approval number P992/12/2019. Ms. Lucy Wambui and Mr. Patrick B. Mutiso (botanists) performed the taxonomic identification, and voucher specimen TEREFE E. /046 was deposited at the United States International University herbarium for future reference.

The dried and powdered plant material was extracted with 1:1 methylene chloride:methanol solvent using the cold maceration technique. Maceration was continued for 7 days with frequent agitation in an orbital shaker, and the resulting liquid was filtered. The extraction was repeated three times, and the filtrates of all portions were pooled in one vessel. Finally, the extract was concentrated using Rota vapor at 30 °C to obtain dry extracts. The resulting mass was weighed and packed in a glass vial and stored in a desiccator over silica gel until use.

### Preparation of solvent partition

The dried crude 1:1 methanol:CH2Cl2 extract of C. dichogamus was dissolved in distilled water (200 mL) and successively partitioned using different solvents of increasing polarity (n-hexane, dichloromethane, ethyl acetate, and methanol) in separatory funnels. The different solvent fractions were then concentrated under reduced pressure using a rotary evaporator and dried in an oven at 30 °C. The dried fractions were then transferred into separate vials and stored in a desiccator for further use.

### In Vitro test

#### Cytotoxicity assay

In this study, human T-lymphocytic MT-4 cells (ARP-120) were obtained through the National Institute of Health (NIH) HIV Reagent Program, Division of AIDS, National Institute of Allergy, and Infectious Diseases (NIAID), NIH: MT-4 Cells, ARP-120, contributed by Dr. Douglas Richman. The cytotoxicity test was conducted to evaluate the plant extracts' safety by measuring cell death caused by the plant extracts. The assay was conducted using the MTT colorimetric assay as described previously [14–17].

#### Anti-HIV activity test

Human immunodeficiency virus type 1 (HIV-1) IIIB (also referred to as HTLV-IIIB) was obtained through the NIH HIV Reagent Program, Division of AIDS, NIAID, NIH: Human Immunodeficiency Virus-1 IIIB, ARP-398, contributed by Dr. Robert Gallo. The effects of the test compounds in preventing the cytopathic effect resulting from HIV-1 replication were evaluated by the MTT colorimetric assay described above. A concentration–response curve was plotted to calculate the concentrations that reduced viral replication by 50% (IC50) [15, 17–19]. The effective concentration at 50% (IC50) is defined as the concentration of the test compound that achieves 50% protection in infected cultures. The selectivity index (SI) of the test compounds was calculated as the ratio of 50% cytotoxic concentration (CC50) to 50% effective concentration (IC50) [17].

#### Statistical analysis

The CC_50_ and IC_50_ values were calculated with GraphPad Prism v9 using a sigmoidal dose–response (variable slope) equation. Statistical significances in comparison between control drugs and extracts cytotoxicity and anti-viral activity parameters (CC_50_, EmaxC, IC_50,_ and Emax AV) were determined by one-way ANOVA followed by Dunnett's post-hoc tests. A difference was considered significant when p < 0.05.

## Results

### Cytotoxicity test

Four solvent fractions of twigs of *C. dichogamus* were examined for their ability to inhibit HIV-1 replication. In addition, the in vitro toxicity of these extracts to human T-lymphocytic MT-4 cells was investigated by MTT assay. The solvent fractions were introduced into the cell cultures at concentrations of 800 – 8.192 × 10^5^ μg/mL, and the optical density (OD) was determined at 540 nm. The average absorbance value from three replicate determinations was used to express the viability of the cells as a percentage of negative controls, and the CC50 for each plant extract was determined graphically by plotting the % growth inhibition (Emax_c_) against the plant extract concentration.

**Fig. 2:**
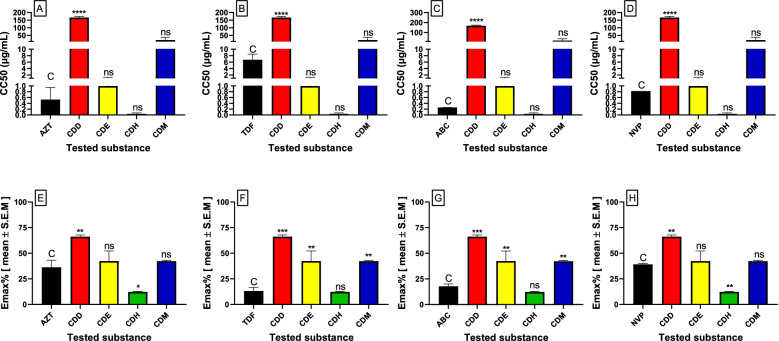
CC_50_ (**A**, **B**, **C** and **D**) and Emax (**E**, **F**, **G** and **H**) values of the control drugs and the tested extracts cytotoxic activity. The results are expressed as the mean of two independent experiments ± S.E.M. C; control, ns; not significant, *; *P* < 0.05 and **; *P* < 0.01, ***; *P* < 0.001, ****; *P* < 0.0001. *AZT*, zidovudine, *ABC*, abacavir, *NVP*, nevirapine, *CDD*, dichloromethane fraction, *CDE*, ethyl acetate fraction, *CDH*, hexane fraction, *CDM*, methanol fraction

As shown in Table 1, the CC_50_ value of the methanol soluble fractions of the 1:1 CH_2_Cl_2_:MeOH extract was 19.58 ± 0.79 µg/mL, indicating that it requires higher concentrations of the fraction for the cytotoxic effect to appear. The methylene chloride soluble fractions of the 1:1 CH_2_Cl_2_:MeOH extract were associated with a significantly (P < 0.01) higher maximum cytotoxic effect by inhibiting more than 60% of cell viability (Emax_C_ = 66.22%), indicating the possibility of higher adverse effects with increasing concentration (Fig. 2).

**Table 1:** Cytotoxicity and anti-HIV-1 effect of control drugs and extracts using the MT4 cell line

Tested substance	Cytotoxicity			Anti-HIV activity		SI
	**MNTC (µg/mL)**	**CC**_**50**_** (µg/mL)**	**Emax**_**C**_** (%)**	**IC**_**50**_** (µg/mL)**	**Emax**_**AV**_** (%)**	
AZT	0.38 ± 0.19	0.53 ± 0.29	36.28 ± 0.83	0.002 ± 0.00	83.5 ± 0.57	279.4
TDF	4.92 ± 0.71	6.73 ± 0.24	13.17 ± 0.43	0.04 ± 0.01	80.55 ± 0.46	176.5
ABC	0.18 ± 0.03	0.26 ± 0.00	17.83 ± 0.57	0.05 ± 0.031	58.67 ± 0.43	5.0
NVP	0.57 ± 0.0	0.82 ± 0.0	39.13 ± 0.65	0.24 ± 0.09	72.53 ± 0.47	3.5
CDD	49.7 ± 0.61	167 ± 0.18	66.22 ± 0.18	0.03 ± 0.02	81.3 ± 0.23	5491.61
CDE	0.84 ± 0.08	0.99 ± 0.2	42.38 ± 0.95	0.55 ± 0.27	77.89 ± 0.99	1.8
CDH	0.02 ± 0.01	0.04 ± 0.02	12.24 ± 0.37	0.03 ± 0.01	77.59 ± 3.52	0.9
CDM	15.4 ± 0.45	19.58 ± 0.79	42.2 ± 0.615	0.06 ± 0.01	90.83 ± 0.18	318.5

*MNTC* (maximum nontoxic concentration), *CC*_50_ (50% cytotoxic concentration), Emax_C_ (maximum cytotoxic effect %), IC_50_ (50% antiviral effect concentration), Emax_AV_ (maximum antiviral effect %), SI (selectivity index). *AZT*, zidovudine, *ABC*, abacavir, *NVP*, nevirapine, *CDD*, dichloromethane fraction, *CDE*, ethyl acetate fraction, *CDH*, hexane fraction, *CDM*, methanol fraction. The results are shown as the means ± S.E.M

Among the FDA-approved antiretroviral drugs, zidovudine and nevirapine showed higher cytotoxicity by inhibiting more than 36% of cell viability compared with tenofovir (Emax_c_ = 13.17%) and abacavir (Emax_C_ = 17.8%).

### Anti-HIV activity test

**Fig. 3:**
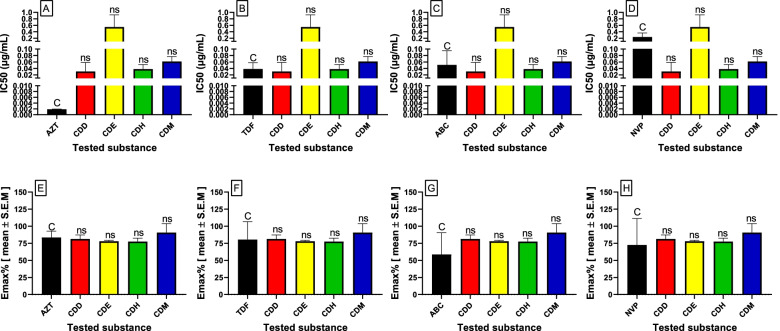
IC_50_ (**A**, **B**, **C** and **D**) and Emax (**E**, **F**, **G** and **H**) values of the control drugs and the tested extracts antiviral activity. The results are expressed as the mean of two independent experiments ± SEM C; control, ns; not significant. *AZT*, zidovudine, *ABC*, abacavir, *NVP*, nevirapine, *CDD*, dichloromethane fraction, *CDE*, ethyl acetate fraction, *CDH*, hexane fraction, *CDM*, methanol fraction

The methanol soluble fractions of the 1:1 CH_2_Cl_2_:MeOH extract of *C. dicohgamus* revealed high anti-HIV activity by inhibiting more than 90% of viral-induced cytopathic effects (Emax_AV_ = 90.83%). The observed viral replication inhibition activity of the methanol soluble fraction with an IC_50_ value of 0.06 ± 0.01 µg/mL giving a selectivity index (SI) of 318.5 was achieved at a much lower concentration than the maximum nontoxic concentration (MNTC = 15.4 ± 0.45 µg/mL), indicating the potency and safety of the extract (Table 1). As shown in Fig. 3, the antiviral effect of the tested extracts was approximately similar to that of the control drugs regarding both their efficacy and potency, as there was no significant difference between their IC_50_ values or Emax_AV_ values of their inhibition of viral-induced CPE**.** Among the FDA-approved antiretroviral drugs, zidovudine showed the highest anti-HIV activity, with IC_50_ values of 0.002 ± 0.00 µg/mL inhibiting 83.5% of CPEs induced by the virus (SI = 279.4), followed by tenofovir, with IC_50_ = 0.04 ± 0.01 and an SI of 176.5.

**Fig. 4:**
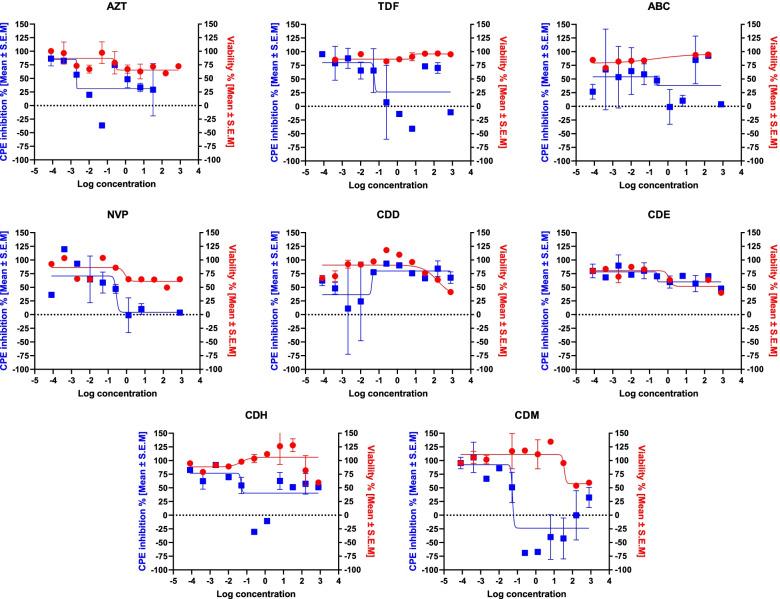
Concentration–response curve analysis for the cell viability % (red line) and the inhibition % of the virus-induced cytopathic effect (blue line) associated with control drugs and the tested extracts at a concentration of 800 – 8.192 × 10^5^ µg/mL. The results presented in the curves are the means ± SEM of three independent experiments

Our findings from the antiviral assay revealed that the ethyl acetate- and hexane-soluble fractions demonstrated little activity against the laboratory-adapted HIV-1 strains. As depicted in Fig. 4, these fractions showed a linear antiviral effect rather than a sigmoidal curve at the concentration level used, which may be due to reaching the maximum effect at a concentration lower than the concentration level used in the experiment. The inactivity of these fractions does not prove that they do not possess anti-HIV1 activity. These fractions can be further investigated for their activity against other viral strains.

Regarding preventing the cytopathic effect of the virus, the methanol soluble fraction showed the best anti-viral activity by inhibiting 90% of the cytopathic effect of the virus on the cell line (IC_50_ = 0.06) compared to the other fractions.

Fractionation of the active methanol soluble fraction led to the isolation and identification of dihydroconiferyl acetate, (4-hydroxy-3-methoxyphenyl)-propyl benzoate, epicrotocascarin M, and *β-opanopanone,* which have been reported in [16].

## Discussion

HIV infection results in the development of acquired immunodeficiency syndrome (AIDS), a clinical syndrome that is caused by a virus called human immunodeficiency virus (HIV), which causes profound immunosuppression. Anti-HIV drugs that are currently available are chemically synthesized, and their effectiveness is frequently limited by side effects and the emergence of drug resistance [20]. Moreover, more than 5 million people [1] still do not have access to the necessary treatment. To address this, it is necessary to identify locally available, less expensive, and less toxic HIV treatment options for the treatment of HIV.

Nature has always been a reliable source of drugs for treating various diseases. Organic compounds, such as those found in plants and animals, continue to be important sources of new anti-infective therapeutic agents. Their investigation has proven to be among the most successful strategies for discovering new medicines. A number of medicinal plants have been found to have anti-HIV properties, according to some reports. Natural products for HIV chemotherapy have been the subject of several reviews [21–24] that have been published previously. Various secondary metabolites derived from natural sources demonstrated moderate to good anti-HIV activity, with some showing excellent activity [21–24].

To find such potential anti-HIV agents from natural sources, solvent fractions of *C. dichogamus* were studied for their inhibitory effects against laboratory-adapted HIV-1_IIIB_ strains in human T-lymphocytic MT-4 cells. In this study, human T-lymphocytic MT-4 cells were used. Harada et al.[25] reported that a lymphocyte cell line, MT-4, which carries the HTLV-I genome, was highly susceptible to HIV infection. MT-4 cells, which are highly susceptible to and permissive for HIV, typically grow in clusters. In the absence of virus, these cell aggregates, after dissociation by pipetting, reform into clusters within 2 to 3 h. After superinfection with HIV, rapid induction and release of HIV antigens were observed, accompanied by a marked cytopathogenic effect [26, 27]. Similarly, many scholars have recently used MT-4 cell lines to evaluate the anti-HIV activity of various compounds [18, 28].

To ensure the safety of the solvent fraction on human T-lymphocytes, a cytotoxicity test was conducted using MT-4 cell lines. Based on our results, the two solvent fractions (CDD and CDM) of twigs of *C. dichogamus* showed a CC_50_ value higher than the control drugs, which indicates that it requires higher concentrations of the fractions for the cytotoxic effect to appear. Among the two fractions, the methanol fraction was relatively nontoxic (Emax_c_ = 42.2%), while the dichloromethane fraction was toxic, causing more than 60% inhibition of cell viability (Table 1). Furthermore, the plant's hexane fraction was cytotoxic at a very low CC_50_ concentration of 0.04 + 0.02 µg/mL.

Our finding on the cytotoxicity of the plant is in agreement with previous reports [9, 29, 30]. Aldhaher et al.[9] reported that 10-epi-manninsigin D, a diterpenoid isolated from the n-hexane fraction of the root of *C. dichogamus*, decreased the viability of Caco-2 human colon carcinoma cell lines by 43% at a concentration of 100 mM. In another study, maninsigin D was cytotoxic with IC_50_ values > 40 mM against HL-60, A-549, SW-480, SMMC-7721, and MCF-7 human cell lines by the MTT method [29].

Cytotoxicity of *C. dichogamus* was also reported by Aldhaher et al*.* [31]. In their report, furocrotinsulolide, a sesquiterpenoid isolated from the methanolic root extract of *C. dichogamus,* showed modest cytotoxicity activity at 30 μm when tested on the Caco-2 cell line [31]. Similarly, in a previous study by Pudhom and Sommit [30], 14-ent-clerodadien-3-one (trans-cascarillone), a clerodane-type diterpenoid isolated from hexane and methanol extract of roots of *C. dichogamus,* showed cytotoxicity against BT-474, KATO-3, CHAGO, SW-620, and HEP-G2 human cancer cell lines with CC_50_ values of 3.26, 6.78, 6.67, 4.44 and 6.37 mg/mL.

The MTT colorimetric assay was used to assess the effectiveness of the test compounds in preventing the cytopathic effect that occurs as a result of HIV-1 replication. The laboratory-adapted strain of human immunodeficiency virus type 1 (HIV-1_IIIB_) obtained from the National Institutes of Health HIV Reagent Program was used in this experiment. A total of 50 µl of different concentrations (800 – 8.192 × 10^5^ μg/mL) of the test compounds were seeded into 96-well flat-bottomed microtiter culture plates containing HIV-infected MT-4 cells.

Our findings from the antiviral assay revealed that the ethyl acetate and hexane fractions of twigs of *C. dichogamus* showed less anti-HIV1 activity against HIV-1_IIIB_. The inactivity of these fractions does not prove that they do not possess anti-HIV-1 activity. These fractions can be further investigated for their activity against other viral strains.

The phytochemical analysis in the current study confirmed the presence of flavonoids, saponins, phenolic compounds, and terpenes in methanol, ethyl acetate, dichloromethane, and hexane fractions of the twigs of *C. dichogamus.* Alkaloids and glycosides were present in the dichloromethane, ethyl acetate, and methanol fractions but were absent in the hexane fractions. Tannins were present in the methanol and ethyl acetate fractions of *C. dichogamus* but were absent in the hexane and dichloromethane fractions.

Regarding preventing the cytopathic effect of the virus, the methanol fraction showed the best antiviral activity by inhibiting 90% of the cytopathic effect of the virus on the cell line (IC50 = 0.06) compared to the other fractions (Fig. 3). The potency of the methanol extract to inhibit the cytopathic effect of the virus could be due to one of the bioactive compounds. A previous study by Aldhaher et al.[9] is in agreement with our phytochemical screening findings for the presence of terpenes in twigs of the fractions, which could have contributed to the observed anti-HIV activity of the methanol fraction. Triterpenoids exhibit antibacterial activities, making the plant useful in treating respiratory infections that are of bacterial origin [32]. Moderate anti-HIV activity was reported for cyanthiwigin B, a diterpene isolated from the Jamaican sponge *Myrmekioderma styx,* in a cell-based in vitro assay. Similarly, other diterpenes, such as betulinic acid, platanic acid and oleanolic acid, isolated from the leaves of *Syzigium claviflorum* have exhibited anti-HIV activity [33].

The potency of the methanol extract to inhibit the cytopathic effect of the virus could also be due to the saponins in the twigs of the plant. In agreement with our phytochemical screening results, Johns et al. [34] reported the presence of high levels of saponins in *C. dichogamus* crude stem extracts with efficacy in lowering cholesterol levels in humans and animals, validating the traditional use of the plant in cardiovascular conditions. Previous studies have shown the efficacy of soybean saponins isolated from soybean seeds in inhibiting HIV-1 replication in MT-4 cells [35]. In another study, acetin, a tetracyclic saponin isolated from the rhizome of *Cimicifuga racemosa* (black cohosh), showed potent anti-HIV activity [36].

## Conclusion

To conclude the study, out of four solvent fractions of *C. dichogamus* screened for anti-HIV activity using human T-lymphocytic MT-4 cells, the methanolic soluble fraction of the twigs of *C. dichogamus* showed potential anti-HIV1 potential as replication inhibitors. Furthermore, since the fractions from twigs of *C. dichogamus* have not been previously analyzed, the methanol fractions could be explored further for activity on different retroviral enzymes. Furthermore, the fraction could be considered for further studies considering the low IC_50_ values compared to CC_50_ values. The margin between the maximum nontoxic concentrations (MNTC) limit and the IC_50_ value was high, confirming its safety.

## Data Availability

The datasets used and/or analyzed during the current study are available from the corresponding author on reasonable request.

## Abreviations

ABC: Abacavir
AZT: Zidovudine
CC50: 50% cytotoxic concentration
CDD: Methylene chloride soluble fractions of 1:1
CH2Cl2: MeOH extract
CDE: Ethyl acetate soluble fractions of 1:1 CH2Cl2: MeOH extract
CDH: : Hexane soluble fractions of 1:1 CH2Cl2:MeOH extract
CDM: methanol soluble fractions of 1:1 CH2Cl2: MeOH extract
EmaxAV: Maximum antiviral effect %
EmaxC: Maximum cytotoxic effect %
IC50: 50% antiviral effect concentration
MNTC: Maximum nontoxic concentration
NVP: Nevirapine
SI: selectivity index
